# Point of care microspirometry to facilitate the COPD diagnostic process in primary care: a clustered randomised trial

**DOI:** 10.1038/s41533-018-0083-9

**Published:** 2018-05-22

**Authors:** Tjard R. Schermer, Maria Vatsolaki, Robbert Behr, Joke Grootens, Riet Cretier, Reinier Akkermans, Joke Denis, Patrick Poels, Lisette van den Bemt

**Affiliations:** 1 Department of Primary and Community Care, Radboud University Medical Center, Nijmegen, Netherlands; 2 Faculty of Medicine, Voutes Campus, Heraklion, Crete, Greece; 3 General practice co-operative OCE, Nijmegen, The Netherlands; 4 Diagnostic Centre Breda SHL, Etten-Leur, Netherlands; 5 General practice Bles and Poels, Huissen, The Netherlands

## Abstract

We studied if pre-bronchodilator FEV_1_/FEV_6_ determinations with microspirometers by GPs improve the diagnostic process for COPD in a 6–8 month clustered randomised controlled trial in Dutch general practices (http://www.trialregister.nl: NTR4041). GPs allocated to microspirometry (MI) used COPD-6^®^ microspirometers in patients ≥50 years old with a smoking history and respiratory complaints that could indicate undiagnosed COPD and ask to refer patients for full spirometry if MI was positive (FEV_1_/FEV_6_ <0.73). Introduction of the COPD-6^®^ was postponed in the usual care (UC) group. GPs of both study arms were asked to list all patients that fulfilled study criteria and at the end of the study we screened the electronic medical record system for number of patients that fulfilled study criteria and visited their GP within the study period. Main end point was a documented diagnostic conclusion of COPD within 3 months after the patient’s visit. We used multilevel logistic regression with correction for relevant covariates. Next, we described the process of care. 21 practices (88 GPs) participated and 416 possible undiagnosed COPD patient visited these practices in the study period. 78 (of 192 visiting) subjects were listed by MI GPs and diagnostic conclusions were documented in 77%, compared to 61 listed (of 224 visiting) subjects and 44% with documented diagnostic conclusions by UC GPs (Odds Ratio: OR: 4.3, 95%CI: 1.6–11.5). Microspirometry improved the diagnostic process for possible underlying COPD in patients who consulted their GP with respiratory symptoms, but the majority of possible undiagnosed COPD patients remained unrecognised by GPs.

## Chronic lung disease: Quick test may help identify potential sufferers

A quick, simple test that can be used by family doctors may help identify patients suffering from chronic obstructive pulmonary disease (COPD). The small, inexpensive microspirometry (MI) kit enables doctors conducting routine appointments to measure the volume of air expelled from patients’ lungs. Lisette van den Bemt at Radboud University Medical Center, the Netherlands, and co-workers worked with two groups of doctors in local practices. Both groups were asked to identify patients over 50 with a smoking history, respiratory problems and no diagnose of asthma and COPD, and start a diagnostic process for COPD. One group was given microspirometers to aid diagnoses. Of 192 patients visiting the MI doctors, 78 were identified and 77 per cent were later listed as COPD or COPD was ruled out. In the other group, 61 out of 224 patients were identified with only 44 per cent listed.

## Introduction

Point of care (POC) tests are innovative diagnostic tests that are carried out on site during a patient visit, with very rapid test results.^1^ POC tests could optimise health care by enhancing diagnostic procedures, improving medical decisions on the one hand, and increase efficiency of care by reducing unnecessary referrals and more advanced diagnostic testing on the other hand. One diagnostic routine in primary care that could benefit from a valid POC test is the detection of chronic obstructive pulmonary disease (COPD).

The hallmark of COPD is a chronic airflow obstruction that is objectified by a post-bronchodilator (BD) spirometry test.^2^ Diagnostic spirometry requires time (usually between 30–45 min) and standardised procedures that cannot be integrated in a regular patient visit to the general practitioners’ (GP) office.^3,4^ GPs consider the lack of objective information during consultation an important barrier in the diagnostic process for COPD.^5^ For instance, it remains difficult to differentiate between a common respiratory infection and COPD in a patient who consults with respiratory symptoms. This is an important reason why COPD is widely underdiagnosed in primary care, with estimates that vary between 45 and 85% among subjects at risk.^6,7^

Forced expiratory volume in 6 s (FEV_6_) is the volume of air that is exhaled in the first 6 s under forced conditions, and can serve as a surrogate for forced vital capacity (FVC).^8^ In the past few years, small and inexpensive handheld devices that measure forced expiratory volume in 1 s (FEV_1_)/ FEV_6_ ratio (microspirometers) have become available. In a previous study, we found that the negative predictive value of a pre-bronchodilator FEV_1_/FEV_6_ <0.73 as measured with a microspirometer to rule out airway obstruction is high in a primary care population at risk of COPD (i.e., 96.3%).^9^ Other studies found comparable results.^10–12^ Thus, microspirometers could help GPs to substantiate airway obstruction more easily and immediately during a regular consultation of an ‘at-risk’ subject. When indicated, the subject can be scheduled for further diagnostic spirometry testing after that. This could facilitate the pathway to an earlier diagnosis of COPD, and at the same time lead to more efficient use of full diagnostic spirometry testing. Studies that focussed on screening (i.e., case finding) for COPD found microspirometry to be a valid method and significantly better than using screening questionnaires.^10,13–15^ But what if microspirometry is used in routine general practice settings as a POC test for COPD? This could potentially reduce underdiagnosis of COPD while avoiding the need for screening initiatives to identify missed COPD cases in primary care populations.

We aimed to study the effect of the introduction of microspirometers in real-life general practice care as a POC test for COPD. We hypothesised that the use of microspirometry would result in a higher proportion of recorded diagnostic conclusions for patients at risk for COPD who visit their GP with respiratory symptoms, compared to usual care conditions.

## Results

**Fig. 1:**
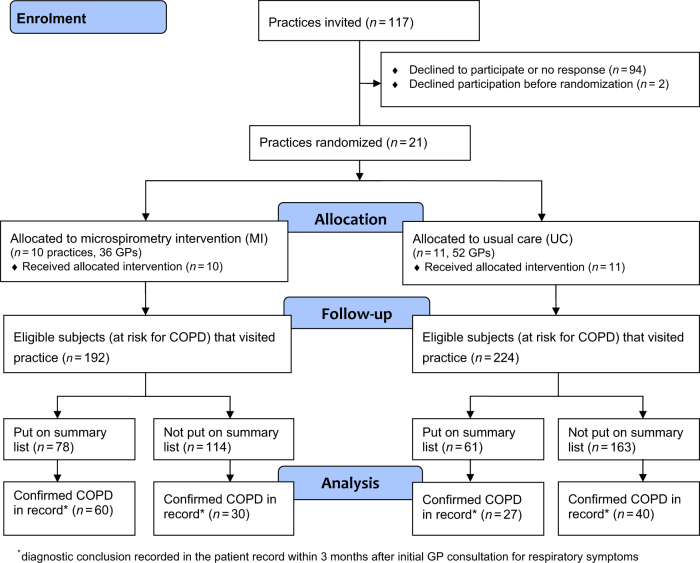
Flow Diagram

This study was carried out in 21 general practices comprising 88 GPs (10 MI practices, Fig. 1). None of the practices dropped out. 14 practices (5 MI) agreed to extent the study period with another two months because of limited numbers of subjects on their summary lists after the initial 6-month period. All but one GP in the MI group passed the exam at the end of the e-learning. The total number of subjects at risk for COPD who visited the practices during follow-up was 416 (192 subjects in MI). There were no subjects who choose to opt-out. Among the subjects who attended the GP office and fulfilled the study criteria, there were more females than males (Table 1). On the basis of the lists generated from the practices’ medical record systems, the number of eligible subjects that visited the practices within the study period varied between 3 and 43 per practice.

**Table 1:** Practice and subject characteristics

	MI group		UC Group	
*General practice level*	*n* = 10 practices		*n* = 11 practices	
Total *n* of GPs	36		52	
GPs per practice, *n* (%)				
≤3 GPs	5	(50)	3	(27)
4–5 GPs	4	(40)	4	(36)
≥6 GPs	1	(10)	4	(36)
Practice population size^a^, mean (SD)	5450	(3017)	6389	(3111)
Diagnosed COPD patients per practice, mean (SD)	101	(54)	111^b^	(63)
Location of practice, *n* (%)				
Urban	3	(30)	9	(82)
Suburban	4	(40)	0	(0)
Rural	3	(30)	2	(18)
Subjects >50 years old, % (SD)	35	(8.1)	29	(7.1)
*Subject leve*l	*n* = 192 subjects		*n* = 224 subjects	
Males, *n* (%)	76	(40)	99	(45)
Age^c^, mean (SD)	64.0	(8.3)	62.7	(7.7)
Smoking habit^d^, *n* (%)				
Current	71	(37)	86	(38)
Former	105	(55)	113	(51)
Unknown	16	(8)	25	(11)

*SD* standard deviation

^a^Number of registered subjects in the practice

^b^Missing information of 1 usual care practice

^c^Age missing for 3 subjects of the usual care group

^d^Subjects with unclear, but existing smoking history were included in the former smokers group

**Fig. 2:**
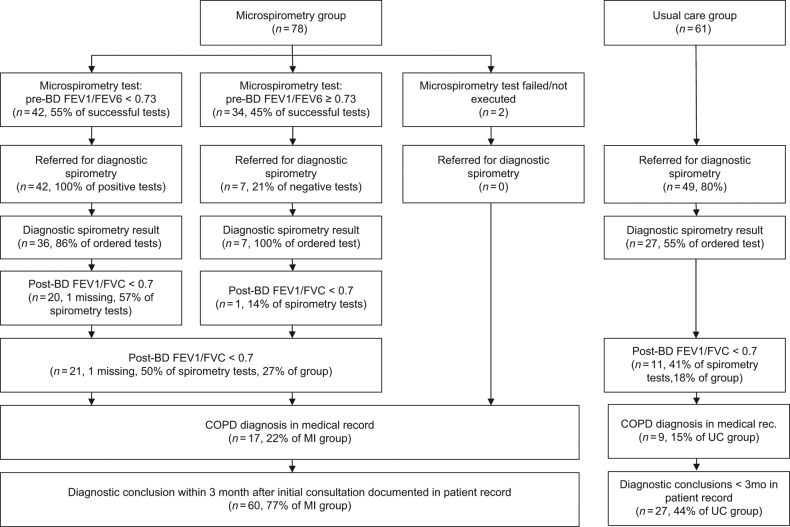
Diagnostic process in subjects on the summary lists of practices in the microspirometry intervention group

More subjects who had visited the practice during the study period and fulfilled the study criteria In the MI group were listed on the practices’ summary lists compared to UC (41 vs. 27%). Figure 2 shows the diagnostic process of subjects that were put on the summary lists in the IM and UC groups in more detail. 57% of the executed diagnostic spirometry tests of subjects with a pre-BD FEV1/FEV6 <0.73 showed airflow obstruction (i.e., post-BD FEV1/FVC < 0.7). Seven subjects were referred for spirometry despite a negative microspirometry test. Only one of these tests showed airflow obstruction. In UC group practices, 29% of diagnostic spirometry tests in subjects on the summary lists were positive. In the appendix (Fig. 1) the diagnostic process of the unlisted patients are visualised.

A diagnostic conclusion was more likely to be drawn within 3 months in the IM group (*n* = 60, 77%) for subjects recorded on practices’ summary lists compared to UC group (*n* = 27, 44%; OR: 4.3, 95%CI: 1.6–11.5). Moreover, 3 patients were diagnosed with asthma in the IM group compared to none in the UC group. No differences between the IM and UC groups were found for subjects that were not listed on the summary lists by the GPs (IM *n* = 34, 30%, UC *n* = 65, 49%; OR: 0.7, 95%CI: 0.3–1.5).

## Discussion

This study, to our knowledge, is the first to look at the impact of introducing microspirometers on the diagnostic process in subjects at risk of COPD in a routine general practice care setting. In subjects who were identified as being at risk for COPD by their GP, the diagnostic process was completed and reported more often when the GP had a microspirometer available. Moreover, the use of diagnostic spirometry appeared to be more efficient in the microspirometry group (i.e., more tests were positive), although the number of subjects for this analysis was small and the difference was not statistically significant. However, no further investigation was initiated in both study groups in the majority of subjects at risk for COPD, despite the fact that they consulted their GP with respiratory symptoms and fit the risk profile for COPD.

The GPs who received the microspirometer seemed to be more aware of COPD compared to GPs that were allocated to usual care, as can be deduced from the higher proportion of eligible subjects that were placed on the summary lists (41 vs. 27%). Moreover, the diagnostic conclusion was more often registered for those who were put on the summary list in the MI group. This observation suggests that POC microspirometry could reduce COPD underdiagnosis in primary care.

Another way to reduce underdiagnosis of COPD is case finding using a screening questionnaire, as has been applied in a recent Dutch study in general practices.^16,17^ Of the 3715 questionnaires that were sent out in this opportunistic primary care case finding strategy, only 48 new COPD cases were found. One of the reasons for this low yield is that only 14% of subjects that filled out the questionnaire were at risk for COPD and could be referred for diagnostic spirometry, while in our study 55% of the microspirometry tests resulted in a referral. Moreover, the attendance rate for diagnostic spirometry testing is higher in our study (86 vs. 54%). A possible explanation for the lower rate of no-show is that the outcome of a microspirometry test is more tangible for patients than a score on a questionnaire. Some studies have compared the results of case finding with a questionnaire and microspirometry head-to-head and conclude that the specificity is much higher for microspirometry and the sensitivity slightly lower or comparable.^15,18^ Moreover, a recent publication showed that the added value of a screening questionnaire next to microspirometry is low.^14^

### Strengths and limitations of our study

We executed a real-life study and analysed the results from a real-life perspective. For example, all spirometry results in the microspirometry group were analysed, including those that were requested by the GP after a negative microspirometry test (i.e., FEV1/FEV6 ≥ 0.73). In daily primary care, GPs can also decide to order a diagnostic spirometry test despite a negative result. Another strength is that, we were the first to include a control group to enable comparison with usual care. Moreover, we verified the documented number of diagnostic conclusions in the subjects’ medical records. This outcome was considered clinically relevant as treatment is guided by confirmed or refuted COPD diagnoses and only documented conclusions are likely to change disease management in individual cases.

A limitation of the study is that GPs may have had good reasons not to put particular subjects on their summary list, reasons that were not recorded in the subjects’ medical records (e.g., terminal ill spouse). The location of the practices (which varied greatly between the two groups) may have influenced findings. Because good access to diagnostic spirometry facilities are available, we do not feel that this has influenced our results. Next, in our study, ‘at risk’ subjects should have smoked at least for three months. Cumulative cigarette smoke exposure is a better indicator, but more difficult to determine. Because of the pragmatic nature of the trial, we decided to ask for months of smoking. Another limitation is that we initially planned to use another definition for ‘diagnostic procedure completed’ in our study protocol. We planned to compare the proportion of patients that had either a confirmed or refuted presence of airflow obstruction. However, only during the data analysis we realised that a negative microspirometry result should also be considered as a refuted airflow obstruction and that because of this, the outcome and intervention were ‘entwined’. The result of the analysis on the original definition of a completed diagnostic procedure is provided in the appendix and shows similar results as the slightly modified outcome that is presented in this paper.

### Implications and recommendations

This study has shown that POC microspirometry is a feasible strategy in routine care that can be implemented without substantial additional time investment or costs in general practices that already have good access to diagnostic spirometry. As microspirometers have originally been developed for single-patient use, it is important to use the more expensive one-way valve mouthpieces in order to prevent cross-infections. Previous studies used another type of microspirometers (like PiKo-6^®^, nSpire Health, Inc) with similar results in test quality, and therefore, it seems that other devices than the COPD-6^®^ can be used for the same purpose.^10,18^ However, little is known about the long-term performance of these devices and the effect of day-to-day use in a general practice. Unlike diagnostic spirometers, microspirometers cannot be calibrated, so more information on the durability of the devices is needed.

## Conclusions

In conclusion, the introduction of POC microspirometry in general practices resulted in more, and more efficient diagnostic spirometry testing in subjects at risk for COPD who consulted their GP with respiratory symptoms. Although microspirometry improved the diagnostic process, the majority of possible undiagnosed COPD patients remained unrecognised by the GPs who were involved in the study.

## Methods

### Study design

We performed a cluster-randomised trial to evaluate the added value of FEV_1_/FEV_6_ measurement using a microspirometer in the diagnostic work-up of subjects at risk for COPD who consult their GP with respiratory complaints. Clusters were general practices in the city of Nijmegen and surroundings, the Netherlands. Subject inclusion started in the first practice in January 2013 and finished in the last practice in December 2014. The study was exempted from ethics review by the medical ethics review board of the Radboud University Medical Center (2012/483). GPs asked permission of subjects to record information for scientific purposes. Study brochures were available in all practices and no identifiable information of patients was collected. The study was conducted according to the principles of the Declaration of Helsinki (Fortaleza, Brazil, October 2013) and Personal Data Protection Act. The study was registered in the Dutch Trial Register (at http://www.trialregister.nl: registration number NTR4041).

### Recruitment of practices and inclusion of study subjects

We invited general practices through primary care cooperatives and an academic general practice network. GPs received an incentive of 250 euro for their participation. Practices could not participate when they had screened their practice population for undiagnosed COPD in the last 5 years and/or were living in a housing development area, where predominantly younger individuals live.

Study participants were subjects who attended a GP in one of the participating practices with respiratory complaints during the study period. The following inclusion criteria applied: 50 years or older; (ex-)smoker with a smoking history of at least 3 months; visited the GP with at least one of the following complaints: dyspnoea, cough, mucus hypersecretion, and/or wheezing; no previous diagnosis of asthma and/or COPD; no diagnostic spirometry test in the past 5 years according to the medical record. Subjects for microspirometry were not preselected with a questionnaire, as filling out a questionnaire by the patient, calculating a score from the questionnaire items and performing microspirometry during a routine GP consultation is not feasible.

### Group allocation and blinding

Practices were allocated to one of the two study arms by means of a minimisation procedure using MINIM software.^19^ Practices were equally distributed for number of registered patients aged 50 + years in the general practice above *versus* equal to or below the Dutch norm. One of the authors (LB) generated the random allocation sequence, and assigned practices to study arms. The allocation sequence was kept at Radboud University Medical Center. Due to the type of intervention the participating GPs could not be blinded for the allocation of their practice. The study’s statistician (RA) was unaware of group allocation during analyses, and the interpretation of results took place before group allocation was revealed.

### Microspirometry intervention and usual care group

GPs in practices allocated to the microspirometry intervention (MI) group followed a 90-min e-learning educational module and instructions on how to use the microspirometry device [COPD-6^®^, Model No 4000, Vitalograph Ltd, Ennis, Ireland] before the start of the study period in their practice.^20^ A total of 3 forced blows into the microspirometer until the stop signal of the device sounded after 6 s were recommended. The microspirometer does not provide any graphical tracings. However, the device does give the operator some feedback about the quality of the forced expiratory manoeuvres during testing. If an exclamation mark appears on the display, this means that the last blow was not a good quality blow and the subject should blow again. GPs were instructed to refer subjects with FEV_1_/FEV_6_ < 0.73 for further diagnostic spirometry testing. MI group GPs were instructed to list all eligible subjects during the study period on a summary list and to use the microspirometer in these subjects when they felt this was indicated and possible timewise.

GPs in the usual care (UC) group did not have access to the e-learning and microspirometers, and were instructed to provide ‘care as usual’ in terms of referring eligible subjects for further diagnostic spirometry testing when they felt this to be indicated. Like in the IM group, UC group GPs were instructed to record subjects who fulfilled the eligibility criteria on the summary list.

The planned duration of the study period per practice was 6 months. However, the number of subjects on the summary lists of some practices was smaller than anticipated, and therefore, we asked these practices to extent the study period with another 2 months.

### Diagnostic spirometric tests

Diagnostic spirometry testing took place in the general practices. Quality of test evaluation and result selection had to be in line with recommendations by international spirometry guidelines.^21^ Pre- and post bronchodilator (BD) spirometric measurements were performed, the latter 15 min after administration of four doses of 100 mcg aerosolized salbutamol by a Volumatic® spacer. Predicted values for FEV_1_ and FVC were calculated based on the Global Lung function Initiative reference equations.^22^

### Data collection

After the study period, each practice printed a list from the electronic medical record system of all subjects who had visited the practice during the study period and fulfilled the following criteria: 50 years or older; respiratory symptoms that could indicate COPD (dyspnoea (International Classification for Primary Care (ICPC) code R02), cough (R05), mucus (R25), and/or wheezing (R03)); no diagnosis of asthma (R96) and/or COPD (R95) on the visiting date; and no spirometry in the past 5 years. For all subjects on the practice list, the following information was extracted manually from their medical files: age; gender; smoking history; information on respiratory diagnostic tests and test results; diagnostic conclusions (also when COPD was ruled out and asthma); if applicable: time between the subject’s initial consultation and the GP’s diagnostic conclusion; and whether or not the subject had also been recorded on the summary list by their GP.

### Outcomes

The primary outcome of the study was the proportion of subjects with a recorded diagnostic conclusion that either confirmed or refuted COPD as evidenced by a lung function test result within 3 months after the initial GP consultation. Apart from this outcome, the process from patient visit till a diagnostic conclusion is described in detail for MI and UC group.

### Sample size calculation

In 2014, the prevalence of COPD was 3.5% in general practices in the Netherlands^23^, and estimates of COPD underdiagnosis in primary care populations vary between 45–85%.^6,7^ A priori we assumed that (a) 30% of subjects that visited their GP and were at risk of COPD would be tested in the UC group and (b) that the use of microspirometry by GPs would double this proportion to 60%. Moreover, we anticipated that at least 10 patients per practice would be listed on the summary list during the study period. Accepting a power of 0.80, α of 0.05 and an intra-cluster correlation of 0.10, and a drop-out of 6 practices, 22 practices had to be recruited.

### Statistical methods

Unpaired *t* tests, *Χ*^2^ tests and Mann–Whitney *U* tests were applied to compare practice, GP and subject characteristics between the IM and UC groups, using IBM SPSS software (Chicago, IL, USA, version 22). Detailed descriptive information on the diagnostic process is provided for both study arms. Logistic regression modelling with correction for clustering on practice level was used to evaluate the difference between MI and UC groups for completed diagnostic process in 3 months after the initial GP consultation (GLIMMIX procedure in SAS statistical software) (SAS Institute, Cary, NC, USA). We tested the model for effect modification and confounding for the following variables: age, gender, smoking status, respiratory symptoms, whether or not the subject had been put on the summary list by the GP, and the follow-up time of the practice (6 vs. 8 months). Because being recorded on the summary list by the GP turned out to be an effect modifier in the analysis, odds ratios (ORs) are presented separately for the comparison of patients on the summary list between IM and UC groups, and for those who had not been put on the summary list by their GPs. For all statistical tests, *p* < 0.05 was considered statistically significant and were tested two-sided.

### Data availability

The data set generated and analysed during the study is currently not publicly available as the Radboud University Medical Center is developing a digital research environment where the data will be made available in the near future (http://portal.umcn.nl/organisatie/im/Pages/Datastewardship.aspx). Until then, the data can be made available by the corresponding author on reasonable request.

## Electronic supplementary material

Supplementary Information
