# Supplementary material for: Point of care microspirometry to facilitate the COPD diagnostic process in primary care: a clustered randomised trial
Source: NPJ Prim Care Respir Med. 2018 May 22;28:17. doi: 10.1038/s41533-018-0083-9 (PMC5964085; doi:10.1038/s41533-018-0083-9)
Supplement: Supplementary file 1 — Supplementary Information [file 41533_2018_83_MOESM1_ESM.pdf]

Figure 1 appendix. Diagnostic process in subjects not on the summary lists of participating microspirometry and usual care practices

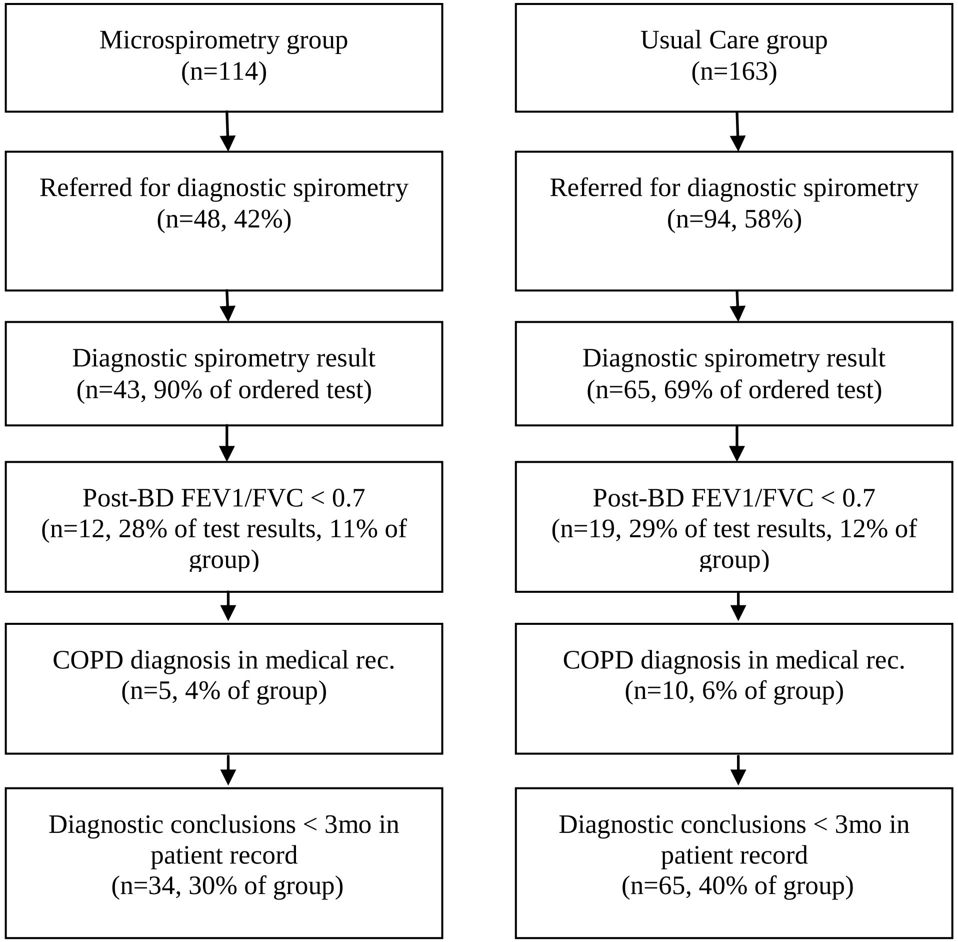

Appendix 2: Results of analysis with original definition of diagnostic process completed and current definition.

	**Microspirometry Intervention (MI) group** (n=192)		**Usual Care (UC) group** (n=224)		**MI *versus* UC**		**MI *versus* UC**	
	Subjects on summary list (n=78)	*Subjects not on summary list (n=114)*	*Subjects on summary list (n=61)*	*Subjects not on summary list (n=163)*	*Subjects on summary list OR (95%CI)*		*Subjects not on summary list OR (95%CI)*	
**Original definition** Diagnostic process completed within 3 mo after consultation, n (%)	65 (83)	43 (38)	37 (61)	80 (49)	3.4	(1.2-9.9)*	0.7	(0.3-1.6)
**Current definition** Diagnostic conclusion within 3 mo after initial consultation documented/ entered in patient record, n (%)	60 (77)	34 (30)	27 (44)	65 (40)	4.3	(1.6-11.5)*	0.7	(0.3-1.5)

* $p < 0.05$
